# Effects of broiler strain and stocking density on causes of death in broilers in Denmark

**DOI:** 10.1016/j.psj.2026.107718

**Published:** 2026-08-27

**Authors:** Tahseen A. AL- Saeedi, Ida C.N. Thøfner, Søren S. Nielsen, Susanne Kabell, Jens P. Christensen

**Affiliations:** a Bacteria and Viruses, Department of Veterinary and Animal Sciences, University of Copenhagen, Frederiksberg C, Denmark; b Department of Animal Production, College of Agriculture, University of AL- Qadisiyah, Iraq; c Animal Health and Welfare, Department of Veterinary and Animal Sciences, University of Copenhagen, Grønnegårdsvej 8, Frederiksberg C, 1870, Denmark; d Rose Poultry part of Plukon, Tværmosevej 10, Vinderup, Denmark

**Keywords:** Rustic gold, Ross 308, Broiler chicken, Stocking density, Mortality

## Abstract

Broiler production industries are increasingly prioritizing animal welfare in line with the traditional focus on production traits such as rapid growth rate and feed efficiency. Broiler strain and stocking density are key welfare factors that are addressed in high-welfare systems. Still, it is unclear how slower-growing broilers or lower densities affect health, causes of death, and gross lesions, which are vital issues in welfare assessment. This study investigated the effects of broiler strain, stocking density, and age on causes of death and gross lesions, with particular attention to growth-related diseases in broilers that died between 7 days of age and slaughter under non-outbreak conditions. It was hypothesized that Rustic Gold broilers would have lower overall mortality and a lower prevalence of growth-related diseases (e.g., cardiovascular diseases and locomotory disorders). Stocking densities and slaughter ages included two Ross 308 broiler densities (33 and 41 kg/m², slaughtered at 34 days) and Rustic Gold broilers at 36 kg/m² (slaughtered at 41 days). The study used a repeated cross-sectional observational design with three rotations per farm/group (36 flocks in total, excluding three). Overall mortality proportions for both Ross 308 groups and Rustic Gold were nearly identical. The data showed that 81% of dead broilers on farms experienced cardiovascular collapse. No significant differences in the primary causes of death, including cardiovascular collapse, leg disorders, renal disorders, septicemia, trauma, polyserositis, cannibalism, and intestinal volvulus, were observed among the different production types. However, Ross 308 exhibited significantly higher prevalences of cardiac dilatation and ascites at both stocking densities than Rustic Gold, whereas the Rustic Gold birds showed higher prevalences of nephropathy and peritonitis. Age analysis showed lower occurrence of cardiovascular collapse and polyserositis in birds ≥28 days, but higher occurrence of leg disorders, renal disorders, and trauma in the same age group.

Overall, from 7 days post-hatch to slaughter age, mortality and the distribution of causes of death in Rustic Gold broilers and Ross 308 at low densities were not significantly different from those in Ross 308 at high density.

## Introduction

Over the past 60 years, selective broiler breeding has primarily targeted production traits such as feed efficiency and growth rate. This focus has led to notable health and welfare issues in meat-producing birds, including cardiovascular problems, leg disorders, and higher mortality proportions (Hartcher and Lum, 2020). Growing consumer concerns have led to a new trend in broiler production, focusing on higher welfare standards by using slower-growing broiler strains, lower stocking densities, and environmental enrichment (de Jong et al*.*, 2022).

In Denmark, approximately 120 million broilers are raised annually, and the proportion of broiler production that includes slower-growing or slow-growing chickens is estimated at 29% (Danish Veterinary and Food Administration, 2025). Reported average growth rates are 62-63 g/day for Ross 308 and 47-51 g/day for Rustic Gold (Riber et al*.*, 2023). Broilers that grow more slowly are reported to have lower mortality proportions than fast-growing ones, possibly because of improved heart health, better mobility, reduced prevalence of contact dermatitis, and greater resistance to heat stress (Riber and Wurtz, 2024).

Danish broiler producers can increase stocking density from 33 kg/m^2^ to 39 kg/m^2^ if they meet environmental and legislative standards, and they may go up to 42 kg/m^2^ if they also satisfy additional criteria, such as no recent animal protection violations, proper management, and low mortality over seven flocks. However, densities above 33 kg/m^2^ must be reported to the competent veterinary authority (European Commission, 2021; Danish Veterinary and Food Administration, 2025). In addition, Riber et al. (2023) reported that the maximum stocking density in Denmark for conventional broilers (Ross 308) was 40 kg/m², and for slower-growing broilers raised under improved welfare and environmental conditions (Ranger Gold or Rustic Gold) was 38 kg/m². In a previous study, a reduction in stocking density was associated with improved comfort, foraging, and play behaviors. Additionally, broilers with slower growth exhibited better movement, comfort, and foraging, and less fear compared to fast-growing broilers (van der Eijk et al*.*, 2022).

A recent study showed that the risk of death after the first week was approximately twice as high in Ross 308 as in Hubbard JA787 (Forseth et al*.*, 2024; Nicol et al*.*, 2024). This contrasts with Torrey et al. (2021), who found no association between growth-rate categories and overall mortality. Currently, fast-growing broilers are more prone to heart disease because their smaller hearts cannot meet the higher circulatory demands of their large bodies (Zhang et al*.*, 2018). The prevalence of tibial dyschondroplasia has been shown to be 2.5 times higher in fast-growing chickens (Shim et al*.*, 2012). Contact dermatitis affected 15-25% more conventional birds, and the average gait score was 0.65 points higher than in slow-growing strains (Nicol et al*.*, 2024). Notably, pododermatitis has been reported to be higher in Ross 308 broilers than in Rustic Gold broilers, with no observed difference in hock burns (Méda et al*.*, 2022). Therefore, under multiple conditions, slower-growing broilers tend to have better health outcomes for various diseases and conditions than fast-growing birds.

The availability of information on the causes of death and specific gross lesions is limited, making these factors valuable indicators of overall welfare. Growth-related diseases are critical in this context. Subsequently, Rustic Gold birds have been approved for use under the RSPCA Assured animal welfare program (Aviagen, 2024). This study aimed to compare mortality, causes of death, and gross lesions in slower-growing (Rustic Gold) and fast-growing (Ross 308) broilers, and to assess the effect of low vs. high stocking density in Ross 308 on these same parameters. The study focused on identifying the causes of death through systematic necropsies. This approach provides valuable insights into disease patterns and welfare-relevant pathological conditions that might not be apparent from mortality data alone. The study was conducted under non-outbreak conditions from 7 days of age to slaughter, adjusting for sex, season, and flock clustering. It also analyzed the impact of age group on causes of death and gross lesions.

## Materials and methods

### Study design

Dead broilers collected from 33 flocks across eight Danish farms were analyzed from 7 days post-hatching until slaughter age as part of a repeated cross-sectional observational study conducted during 2023–2024. The study was considered a repeated cross-sectional design because dead broilers were sampled repeatedly from the same commercial flocks, with each necropsied bird representing a different individual rather than repeated observations of the same birds. From 7 days of age until slaughter, farmers collected the first four broilers found dead naturally each morning on weekdays (Monday to Friday) from each flock, without selection based on carcass appearance or suspected cause of death. If available, one euthanized bird was also included. In total, 2,763 broilers were collected. Carcasses were stored at −20°C on the farms before being transported frozen to the Department of Veterinary and Animal Sciences at the University of Copenhagen, where they remained at −20°C until necropsy.

A slower-growing broiler strain (Rustic Gold) was raised at a density of 36 kg/m² on four farms (A-D). Meanwhile, a fast-growing strain (Ross 308) was raised at two stocking densities (low, 33 kg/m²; high, 41 kg/m²) in a rotating order across four farms (E-H). The analyses covered three rotations, one in each season: summer, autumn, and winter, except for farm E, which only supplied birds from two seasons of each Ross 308 group. Similarly, farm D also provided birds from only two seasons. The eight farms were located on the island of Funen and the Jutland peninsula in Denmark. The average slaughter age was 34.2 days (range 32-37) for Ross 308-HD flocks, 33.8 days (range 31-36) for Ross 308-LD flocks, and 41.3 days (range 40-43) for Rustic Gold flocks. All flocks originated from the same commercial hatchery and were processed at the same commercial slaughterhouse. Litter material consisted of peat (Spagnum), wood shavings, or a combination of both, and feed was supplied by commercial Danish feed manufacturers. All flocks were vaccinated except for four Rustic Gold flocks, although information on the specific vaccines administered was not available. All flocks, except for one Ross 308-LD from farm G, were not treated with antibiotics. First-week mortality (FWM) was excluded from the analysis because it is considered a separate entity. The conditions affecting FWM are likely influenced by factors such as parent stock, hatchery conditions, and neonatal management (Yassin et al*.*, 2009; Yerpes et al*.*, 2020; Swelum et al*.*, 2021).

### Post-mortem examination

All necropsies were conducted in accordance with established protocols by two experienced poultry pathologists (15 and 30 years of experience, respectively). A total of 40 macroscopic observations across all organ systems were recorded at the bird level. In each bird, a tentative cause of death was noted based on a comprehensive evaluation of gross lesions and likely pathogeneses (Table 1). In some cases, bacteriology was needed to confirm the cause of death. The slaughterhouse provided mortality data for the production period across the eight farms (33 flocks).

**Table 1:** Definitions of pathological findings recorded in broilers aged >7 days.

Pathological findings	Definition
Cardiovascular collapse	Defined as a composite pathological diagnosis based on the concurrent presence of ≥3 gross lesions indicative of circulatory failure and/or acute cardiac dysfunction, including ventricular dilatation, ascites, pulmonary edema, subcutaneous edema, mucosal congestion and hyperemia of the trachea with or without catarrhal exudate, generalized circulatory disturbances, liver cirrhosis, and serous effusion.
Leg disorders	Arthritis and/or epiphysiolysis and/or osteomyelitis and/or femoral head necrosis and/or tibial dyschondroplasia and/or leg fractures
Renal disorders	Nephropathy (increased tubular pattern and/or renomegaly and/or urate in ureters and/or pale kidneys), possibly urate deposits in other organ systems and/or joints (visceral gout with or without uremic muscle and dehydration).
Septicemia	Focal or diffuse necrosis of the liver and/or spleen, and/or hepatomegaly and/or splenomegaly, and/ or if bacteria are isolated from the spleen and/or liver.
Trauma	Intra-abdominal hemorrhage (such as liver rupture or renal and other site involvement) and/or fractures of any bone.
Polyserositis	Fibrinous or fibrinopurulent inflammation of two or more serous membranes, including pericarditis, perihepatitis, peritonitis, and airsacculitis.
Intestinal volvulus	This condition involves twisting of an intestinal segment around its mesentery, causing obstruction, with gas- or fluid-filled segments before the twist, a collapsed segment after, venous congestion, and possible necrosis.
Cannibalism and/or pecking	Pecking, especially in the cloacal region, and/or ante-mortem cannibalism (evisceration)
Skin lesions	Involve one or more of the following conditions: scratches, wounds, cellulitis, footpad dermatitis, hock burn, bruises, or subcutaneous bleeding.

### Bacteriological examination

Bacterial culture was performed on non-autolytic broilers exhibiting macroscopic signs of infection, including exudates on serosal membranes; necrosis of the bone marrow, liver, and/or spleen; hyperemia; or organ swelling, either localized or widespread. The organ's surface was sterilized with a burning hot iron. A sterile cotton swab was used to sample the organ, which was then streaked onto a blood agar plate containing 5% calf blood (Oxoid, Basingstoke, United Kingdom). One or more samples were taken from each bird, depending on whether the condition was deemed localized or systemic. The plate was incubated aerobically overnight at 37 °C (Poulsen et al*.*, 2017). Bacterial isolates were identified at the species level using Matrix-Assisted Laser Desorption Ionization–Time of Flight (MALDI-TOF) Mass Spectrometry (Vitek MS; bioMérieux, Ballerup, Denmark) (Karunarathna et al*.*, 2017).

### Data management

Data obtained from necropsies were entered into a Microsoft Excel© spreadsheet. Each tentative independent cause of death or each lesion was recorded as either present or absent. A single broiler could have multiple independent tentative causes of death. The raw data were organized by assigning unique numbers to each variable and their categories, such as broiler production types, including Rustic Gold, Ross 308-LD, and Ross 308-HD, age groups (<18 days, 18-28 days, and ≥ 28 days), sex (male and female), season (summer, autumn, winter), and flock (Flock ID 1 to 33).

Bacterial isolates were documented in a separate Microsoft Excel© spreadsheet by sampling location (liver, skin, spleen, bone marrow, joint, pericardium, and peritoneum) to confirm suspected pathological diagnoses. They were categorized as either pure or mixed cultures based on the predominant isolate. From this, a separate Microsoft Excel® spreadsheet was created for the 240 bacterial isolates identified by MALDI-TOF, with one bacterial species per bird, and the outcomes were recorded as binary (0 or 1). The bacterial data were organized by assigning unique numbers, such as broiler production types (Rustic Gold, Ross 308-LD, and Ross 308-HD), or sampling location (liver, bone marrow, joint, and other organs (skin, spleen, pericardium, and peritoneum).

### Statistical analysis

To display the data, analyses, and visualizations, R (R Core Team, 2024) was used. Mortality was evaluated as a binomial outcome, calculated as the number of dead broiler chickens from 7 days post-hatch until slaughter age, divided by the total broilers on the farm at 7 days old, within each group. A quasi-binomial generalized linear model was used for analyzing mortality data. Pairwise comparisons were obtained through model-based contrasts.

The primary explanatory variable was the broiler production type (Rustic Gold, Ross 308-LD, and Ross 308-HD). Each necropsy outcome was treated as a binary variable (presence/absence). Frequencies and proportions of causes of death and related gross lesions were initially calculated as descriptive statistics for broiler strain and stocking density groups (Rustic Gold, Ross 308-LD, and Ross 308-HD), and for age groups (<18 days, 18-28 days, and ≥28 days).

Poisson regression with robust variance estimation was used because it yields accurate estimates, is more interpretable, and is preferable to logistic regression for analyzing cross-sectional studies with binary outcomes (Barros and Hirakata, 2003). Therefore, the effects of the production type (Rustic Gold as the reference) and age categories (<18 days as the reference) on cause of death and gross lesion prevalence were estimated using multivariable Poisson regression models with a log link and cluster-robust standard errors (Huber–White sandwich estimator). This method directly estimates adjusted prevalence ratios (PR) and 95% confidence intervals. Since the analysis did not include live birds besides dead birds, the ratios reflect the probabilities of causes of death rather than population risks. All models included two a priori confounders, sex and season, and flock as a clustering variable in the variance estimation because birds within the same flock shared housing, management, and environmental factors; therefore, observations were not independent. Generalized linear mixed models with flock as a random intercept were also fitted as part of model validation to assess convergence, random-effect variance, and residual diagnostics. Together with the remaining diagnostic procedures, these assessments supported the adequacy of the primary modeling approach.

Adjusted prevalence ratios and 95% confidence intervals were calculated for three pairwise comparisons: (Rustic Gold vs Ross 308-LD, Rustic Gold vs Ross 308-HD, and Ross 308-LD vs Ross 308-HD) in the first sub-analysis and (<18 days vs 18-28 days, <18 days vs ≥ 28 days, and 18-28 days vs ≥ 28 days) in the second sub-analysis. For each outcome, *p*-values were obtained through Wald tests with cluster-robust standard errors. The Benjamini–Hochberg FDR correction was applied to control the false-positive rate. Statistical significance was determined at an FDR-adjusted overall *p* < 0.05.

Model diagnostics indicated good performance, with no evidence of multicollinearity, separation, or problematic overdispersion, and satisfactory convergence across outcomes. Differences between crude and adjusted estimates were minor, suggesting minimal confounding and enhanced robustness of inference.

### Ethical considerations

The project was approved by the University of Copenhagen’s Department of Veterinary and Animal Sciences’ Animal Ethics Institutional Review Board (AEIRB) (ref. no. 2023-02-VCM-004A).

## Results

This study was designed to compare mortality, causes of death, and gross lesions in slower-growing and fast-growing broilers, and to assess the effect of low vs. high stocking density in Ross 308. It also analyzed the impact of age group on causes of death and gross lesions. A total of 2,508 broiler chickens were necropsied, while 255 broilers were deemed unfit for necropsy due to autolysis. The necropsied and unfit birds were collected from the 17,796 birds that died on eight farms (33 flocks) ≥ 7 days of age (Table 2). The average slaughter ages were 34 days (range 32-37) for Ross 308-HD, 31-36 for Ross 308-LD, and 41.3 days (40.4-42.6) for Rustic Gold. The overall mortality and range of mortality for this period were 2.17% (range 1.81-2.81%) for Rustic Gold flocks, 2.31% (range 1.44-4.60%) for Ross 308-LD flocks, and 1.92% (range 1.11-2.66%) for Ross 308-HD flocks. Of all the dead broilers during the study period, 13.9% of Rustic Gold, 14.5% of Ross 308-LD, and 14% of Ross 308-HD birds were necropsied. The quasi-binomial generalized linear model showed no statistically significant difference in the overall mortality proportion ≥ 7 days post-hatch among the Rustic Gold, Ross 308-LD, and Ross 308-HD groups (*F* = 0.50, *p* = 0.61). In addition, pairwise comparisons confirmed no significant differences among any production comparisons.

**Table 2:** Distribution of necropsied birds, mortality after the first week, and total mortality proportions across eight farms (33 Flocks).

Group	No. of Flocks	Necropsied & Mortality %	Farm A No of Dead Broilers (%)	Farm B No of Dead Broilers (%)	Farm C No of Dead Broilers (%)	Farm D No of Dead Broilers (%)	Total Number, (mean %, and range %)
Rustic Gold	11	Necropsied dead Birds	282 (14.3%)^a^	256 (16.2%)	277 (15.2%)	186 (10.2%)	1001 (13.9%, range 10.2-16.2%)
		Mortality > 7 days old	(1.81-2.81%) ^b^	(1.86-2.25%)	(2.05-2.44%)	(1.91-2.32%)	7200 (2.17%, range 1.81-2.81%)
		Overall Mortality	(3.08-4.48%)	(2.83-3.15%)	(2.50-4.29%)	(2.65-3.03%)	10639 (3.19%, range 2.50-4.48%)
			Farm E No of Dead Broilers ^c^ (%)	Farm F No of Dead Broilers (%)	Farm G No of Dead Broilers (%)	Farm H No of Dead Broilers (%)	

Ross 308-LD	11	Necropsied dead Birds	142 (17.6%)	216 (16%)	232 (19.5%)	164 (8.8%)	754 (14.5%, range 8.8-19.5%)
		Mortality >7 days old	(1.44-1.93%)	(1.85-2.29%)	(2.02-4.60%)	(1.47-2.44%)	5211 (2.31%, range 1.44-4.60%)
		Overall Mortality	(2.52-2.77%)	(2.78-3.52%)	(2.67-5.73%)	(2.07-3.26%)	6684 (3.25%, range 2.07-5.73%)
Ross 308-HD	11	Necropsied dead Birds	143 (14.1%)	205 (15%)	224 (19.8%)	181 (9.7%)	753 (14%, range 9.7-19.8%)
		Mortality >7 days old	(1.11-2.24%)	(1.55-1.98%)	(1.94-2.65%)	(2.13-2.66%)	5385 (1.92%, range 1.11-2.66%)
		Overall Mortality	(2.03-4.06%)	(2.13-2.68%)	(2.94-3.61%)	(2.87-3.49%)	8301 (2.76%, range 2.03-4.06%)

a The number shows the total number of necropsied birds across three rotations on a farm, while the proportion indicates the sample fraction. b The proportions represent the highest and lowest mortality observed from more than 7 days up to slaughter age across three broiler rearing rotations on a farm. c Each farm participated in raising broilers for three rotations, except for farms D and E, which provided two rotations of each Ross 308 group.

### Distribution of causes of death in found-dead and euthanized broilers

The proportion of euthanized broilers in the total sample was 311 (12.4%): 14.69% for Rustic Gold birds, 11.4% for Ross 308-LD, and 10.36% for Ross 308-HD. Birds were euthanized by farmers when they showed severe clinical signs related to ascites, septicemia, polyserositis, or uremia (42.12%), as well as lameness (36.33%), emaciation (14.15%), and trauma or cannibalism (2.61%). Necropsies were performed on these euthanized birds and on broilers found dead (*n* = 2197, 87.6%) to determine causes of death. Because some birds had multiple concurrent possible causes of death, they were classified into more than one cause-of-death category. Consequently, the cumulative proportions across all causes of death exceed 100%.

### Causes of death by production type

In the Poisson regression model with a log link, adjusted for sex and season, and with standard errors clustered at the flock level, the overall p-adjusted effect estimates for the most important causes of death, and their associated gross lesions, are presented (Table 3).

**Table 3:** displays the distribution of causes of death and their associated gross lesions in dead broiler groups aged over 7 days: Rustic Gold (*n* = 1001), Ross 308-LD (*n* = 754), and Ross 308-HD (*n* = 753). The table comprises three sections: descriptive statistics (counts and proportions), overall adjusted *p*-values, and adjusted prevalence ratios (PRs) for three comparisons across all binary outcomes examined.

Pathological findings	Overall occurrence	Rustic Gold	Ross 308-LD	Ross 308-HD	Overall p adj (BH)*	PR RG vs LD (95% CI)	PR RG vs HD (95% CI)	PR LD vs HD (95% CI)
**Cardiovascular Collapse (overall)**⁎⁎	**2034 (81.2%)**	**756 (75.6%)**	**629 (83.8%)**	**649 (86.2%)**	**0.124**	**1.12 (1.01–1.24)**	**1.15 (1.03–1.29)**	**0.97 (0.91–1.04)**
Cardiovascular Collapse with cardiac dilatation	718 (28.7%)	201 (20.1%)	256 (34.1%)	261 (34.7%)	0.027	1.72 (1.23–2.39)	1.74 (1.17–2.59)	0.99 (0.73–1.32)
Cardiovascular Collapse without cardiac dilatation	1316 (52.5%)	555 (55.4%)	373 (49.5%)	388 (51.5%)	0.643	0.90 (0.77–1.06)	0.94 (0.78–1.13)	0.96 (0.81–1.14)
Muscular (skeletal) Discoloration ⁎⁎⁎	915 (36.5%)	320 (32%)	307 (40.7%)	288 (38.2%)	0.242	1.29 (1.01–1.64)	1.19 (0.97–1.45)	1.08 (0.89–1.33)
Generalised Circulatory Disturbances	1901 (76.1%)	703 (70.4%)	597 (79.7%)	601 (79.9%)	0.136	1.16 (1.02–1.31)	1.17 (1.02–1.33)	0.99 (0.91–1.08)
Lung Edema Congestion	2051 (82.0%)	755 (75.5%)	637 (84.8%)	659 (87.7%)	0.124	1.13 (1.00–1.28)	1.17 (1.03–1.33)	0.97 (0.91–1.03)
Tracheal Changes	1790 (73.0%)	649 (65.5%)	569 (78.2%)	572 (78.1%)	0.055	1.21 (1.06–1.38)	1.21 (1.05–1.41)	1.00 (0.91–1.10)
Subcutaneous Edema	2001 (80.1%)	726 (72.7%)	624 (83.3%)	651 (86.8%)	0.024	1.16 (1.04–1.29)	1.20 (1.08–1.34)	0.96 (0.91–1.02)
Ascites	185 (7.4%)	47 (4.7%)	76 (10.1%)	62 (8.3%)	0.024	2.12 (1.37–3.30)	1.72 (0.97–3.05)	1.23 (0.73–2.08)
Liver Cirrhosis	53 (2.1%)	13 (1.3%)	24 (3.2%)	16 (2.1%)	0.124	2.30 (1.20–4.41)	1.47 (0.58–3.74)	1.56 (0.59–4.14)
Pericardial Serous Effusion	84 (3.4%)	27 (2.7%)	14 (1.9%)	43 (5.7%)	0.331	0.70 (0.26–1.93)	2.07 (0.82–5.26)	0.34 (0.10–1.12)
**Leg Disorders**	**257 (10.3%)**	**120 (12%)**	**58 (7.7%)**	**79 (10.5%)**	**0.511**	**0.63 (0.34–1.15)**	**0.85 (0.49–1.47)**	**0.74 (0.40–1.38)**
Arthritis	136 (5.4%)	70 (7%)	33 (4.4%)	33 (4.4%)	0.400	0.57 (0.27–1.22)	0.57 (0.27–1.21)	1.01 (0.43–2.36)
Epiphysiolysis	119 (4.8%)	54 (5.4%)	20 (2.7%)	45 (6%)	0.107	0.51 (0.25–1.07)	1.15 (0.61–2.18)	0.45 (0.24–0.83)
Femoral Head Necrosis	117 (4.7%)	55 (5.5%)	24 (3.2%)	38 (5.1%)	0.462	0.59 (0.28–1.23)	0.92 (0.46–1.84)	0.63 (0.33–1.23)
Osteomyelitis	99 (4.0%)	46 (4.6%)	16 (2.1%)	37 (4.9%)	0.170	0.49 (0.23–1.04)	1.11 (0.62–1.97)	0.44 (0.22–0.90)
Tibial Dyschondroplasia	108 (4.3%)	49 (4.9%)	32 (4.3%)	27 (3.6%)	0.891	0.93 (0.53–1.66)	0.77 (0.36–1.68)	1.21 (0.60–2.43)
**Renal Disorders**	**140 (5.6%)**	**68 (6.8%)**	**36 (4.8%)**	**36 (4.8%)**	**0.623**	**0.71 (0.39–1.29)**	**0.70 (0.32–1.52)**	**1.01 (0.43–2.41)**
Nephropathy	161 (6.4%)	95 (9.5%)	28 (3.7%)	38 (5.1%)	0.022	0.40 (0.24–0.69)	0.55 (0.29–1.03)	0.74 (0.37–1.47)
Renomegaly	59 (2.4%)	18 (1.8%)	21 (2.8%)	20 (2.7%)	0.725	1.61 (0.60–4.31)	1.49 (0.40–5.52)	1.08 (0.29–4.01)
Uremic Muscles	68 (2.7%)	48 (4.8%)	12 (1.6%)	8 (1.1%)	< 0.001	0.32 (0.18–0.57)	0.21 (0.12–0.39)	1.50 (0.82–2.75)
Dehydration	133 (5.3%)	84 (8.4%)	19 (2.5%)	30 (4%)	0.003	0.30 (0.16–0.56)	0.48 (0.28–0.84)	0.63 (0.31–1.27)
Gout	9 (0.4%)	3 (0.3%)	3 (0.4%)	3 (0.4%)	0.985	1.16 (0.24–5.68)	1.13 (0.21–6.11)	1.03 (0.27–3.93)
**Septicemia**	**112 (4.5%)**	**52 (5.2%)**	**23 (3.1%)**	**37 (4.9%)**	**0.306**	**0.61 (0.37–1.03)**	**0.97 (0.53–1.76)**	**0.63 (0.33–1.21)**
Liver Necrosis	39 (1.6%)	17 (1.7%)	6 (0.8%)	16 (2.1%)	0.417	0.49 (0.17–1.40)	1.28 (0.53–3.08)	0.38 (0.12–1.18)
Hepatomegaly	120 (4.8%)	55 (5.5%)	23 (3.1%)	42 (5.6%)	0.668	0.57 (0.21–1.53)	1.02 (0.38–2.73)	0.56 (0.18–1.75)
Splenomegaly	37 (1.5%)	21 (2.1%)	3 (0.4%)	13 (1.7%)	0.051	0.19 (0.06–0.57)	0.81 (0.30–2.19)	0.23 (0.06–0.89)
**Trauma**	**101 (4.0%)**	**39 (3.9%)**	**32 (4.3%)**	**30 (4%)**	**0.985**	**1.01 (0.60–1.68)**	**0.95 (0.54–1.70)**	**1.06 (0.52–2.13)**
**Polyserositis**	**94 (3.8%)**	**52 (5.2%)**	**18 (2.4%)**	**24 (3.2%)**	**0.206**	**0.49 (0.26–0.93)**	**0.65 (0.32–1.33)**	**0.76 (0.41–1.41)**
Pericarditis	102 (4.1%)	56 (5.6%)	20 (2.7%)	26 (3.5%)	0.278	0.51 (0.26–0.99)	0.66 (0.31–1.40)	0.77 (0.39–1.50)
Perihepatitis	87 (3.5%)	45 (4.5%)	19 (2.5%)	23 (3.1%)	0.425	0.59 (0.31–1.10)	0.71 (0.36–1.39)	0.83 (0.45–1.53)
Peritonitis	72 (2.9%)	46 (4.6%)	11 (1.5%)	15 (2%)	0.047	0.34 (0.17–0.71)	0.47 (0.23–0.93)	0.74 (0.36–1.53)
Airsacculitis	68 (2.7%)	35 (3.5%)	14 (1.9%)	19 (2.5%)	0.591	0.56 (0.24–1.32)	0.77 (0.31–1.90)	0.73 (0.34–1.57)
**Intestinal Volvulus**	**47 (1.9%)**	**19 (1.9%)**	**11 (1.5%)**	**17 (2.3%)**	**0.891**	**0.80 (0.20–3.16)**	**1.23 (0.28–5.45)**	**0.65 (0.18–2.32)**
**Cannibalism and Pecking**	**39 (1.6%)**	**31 (3.1%)**	**3 (0.4%)**	**5 (0.7%)**	**0.055**	**0.12 (0.02–0.67)**	**0.20 (0.05–0.74)**	**0.60 (0.11–3.40)**
**Non Specific Lesion**	**41 (1.6%)**	**12 (1.2%)**	**15 (2%)**	**14 (1.9%)**	**0.702**	**-**	**-**	**-**
**Skin Lesions**	**305 (12.2%)**	**123 (12.3%)**	**50 (6.6%)**	**132 (17.5%)**	**0.011**	**0.53 (0.32–0.88)**	**1.38 (0.70–2.70)**	**0.38 (0.22–0.68)**
Scratches and Wounds	179 (7.2%)	94 (9.4%)	22 (2.9%)	63 (8.4%)	0.003	0.30 (0.16–0.57)	0.86 (0.33–2.25)	0.35 (0.15–0.82)
Hock Burn	75 (3.0%)	13 (1.3%)	15 (2%)	47 (6.3%)	0.043	1.50 (0.43–5.22)	4.71 (1.35–16.42)	0.32 (0.13–0.76)
Footpad Dermatitis	34 (1.4%)	3 (0.3%)	4 (0.5%)	27 (3.6%)	0.043	1.88 (0.16–22.26)	12.26 (1.40–106.94)	0.15 (0.03–0.72)
Bruises	29 (1.2%)	10 (1%)	8 (1.1%)	11 (1.5%)	0.926	0.91 (0.25–3.26)	1.21 (0.32–4.59)	0.75 (0.22–2.53)
Subcutaneous Bleeding	21 (0.8%)	9 (0.9%)	7 (0.9%)	5 (0.7%)	0.724	0.87 (0.37–2.05)	0.66 (0.30–1.47)	1.33 (0.47–3.72)
Cellulitis	28 (1.1%)	14 (1.4%)	6 (0.8%)	8 (1.1%)	0.702	0.61 (0.25–1.47)	0.83 (0.36–1.89)	0.74 (0.29–1.88)

*p*-values were adjusted using the Benjamini–Hochberg procedure.

⁎ indicates adjusted *p* < 0.001.

⁎⁎ Causes of mortality after the first week in bold.

⁎⁎⁎ Only discoloration associated with cardiovascular collapse was recorded, such as congestion, pallor with congestion, and generalized cyanosis.

Among dead birds with recorded causes of death, cardiovascular collapse was observed in 2034 (81.2%) cases. It was diagnosed in 86.2% of Ross 308-HD, 83.8% of Ross 308-LD, and 75.6% of Rustic Gold birds, making it the leading cause of death. There were no significant differences among the groups regarding cardiovascular collapse. However, both Ross 308 groups showed higher proportions of birds experiencing cardiovascular collapse accompanied by cardiac dilatation and subcutaneous edema than Rustic Gold birds, with prevalence ratios and 95% confidence intervals (CIs) of 1.72 (1.23–2.39) and 1.16 (1.04–1.29) for Ross 308-LD vs Rustic Gold birds and 1.74 (1.17–2.59) and 1.20 (1.08–1.34) for Ross 308-HD vs Rustic Gold. In addition, ascites was higher in Ross 308-LD than in Rustic Gold, with a prevalence ratio of 2.12 (1.37–3.30).

A total of 257 dead birds (10.3%) had at least one leg disorder. This was the second most common cause of death and euthanasia, accounting for 10.5% in Ross 308-HD, 7.7% in Ross 308-LD, and 12% in Rustic Gold birds. However, there were no statistically significant differences between the groups when comparing leg disorders, whether on a per-bird basis (since a bird could have multiple leg lesions) or based on specific gross lesions (considering each leg lesion individually).

A total of 140 (5.6%) dead broilers on farms exhibited an overall pattern of renal disorders. This was the third most common cause of death across all groups, representing 4.8% in each of the Ross 308 groups and 6.8% in Rustic Gold birds. There were no significant differences among the groups regarding the overall renal disorder pattern. However, the prevalence of nephropathy, dehydration, and uremic muscles in the Rustic Gold group was more than 2.5 times higher: prevalence ratios and 95% CIs of 2.50 (1.45–4.17), 3.13 (1.75–5.56), and 3.33 (1.79–6.25), respectively, compared with Ross 308-LD. Additionally, the prevalence of uremic muscle in the Rustic Gold group was more than four and a half times higher than in Ross 308-HD (PR = 4.76, 95% CI: 2.56–8.33).

A total of 112 (4.5%) birds died due to septicemic conditions. This was the fourth most common cause of death across all groups, accounting for 4.9% in Ross 308-HD, 3.1% in Ross 308-LD, and 5.2% in Rustic Gold birds, with no significant differences among groups.

Trauma was observed in 101 (4%) of dead broilers, making it the fifth most common cause of death across all groups. It accounted for 4% in Ross 308-HD, 4.3% in Ross 308-LD, and 3.9% in Rustic Gold birds, with no significant differences among groups.

Polyserositis was observed in 94 dead broilers on farms, representing 3.8% of cases, and was the sixth most common cause of death. It accounted for 3.2% in Ross 308-HD, 2.4% in Ross 308-LD, and 5.2% in Rustic Gold birds. No significant differences in polyserositis or related gross lesions were observed, except for one comparison showing a statistically significant difference, in which the relative probability of peritonitis was more than 3 times higher in the Rustic Gold group than in the Ross 308-LD group (PR=3.23, 95% CI: 1.52–6.67).

### Causes of death by age group

To evaluate the association between age and causes of death, birds were categorized into three age groups (<18 days, 18–28 days, and ≥28 days). All birds were included in the analysis regardless of strain or stocking density (Table 4).

**Table 4:** displays the distribution of causes of death and their associated gross lesions across three age groups: < 18 days (*n* = 927), 18-28 days (*n* = 900), and ≥ 28 days (*n* = 680). The table consists of three sections: descriptive statistics (numbers and proportions), overall adjusted *p*-values, and adjusted prevalence ratios (PR) for these comparisons across all binary outcomes examined.

Pathological finding	<18 Days	18_28 Days	≥28 Days	Overall p adj (BH)*	PR <18 vs 18-28 Days (95% CI)	PR <18 vs ≥28 Days (95% CI)	PR 18-28 vs ≥28 Days (95% CI)
**Cardiovascular Collapse (overall)**⁎⁎	**793 (85.7%)**	**777 (86.3%)**	**464 (68.3%)**	**< 0.001**	**1.00 (0.94–1.06)**	**0.79 (0.70–0.89)**	**1.27 (1.15–1.39)**
Cardiovascular Collapse with Cardiac Dilatation	215 (23.2%)	269 (29.9%)	234 (34.5%)	0.059	1.28 (0.94–1.75)	1.50 (1.08–2.08)	0.85 (0.68–1.07)
Cardiovascular Collapse without Cardiac Dilatation	578 (62.3%)	508 (56.4%)	230 (33.8%)	< 0.001	0.90 (0.79–1.03)	0.54 (0.46–0.62)	1.68 (1.48–1.91)
Lung Edema Congestion	794 (85.8%)	785 (87.2%)	472 (69.7%)	< 0.001	1.01 (0.96–1.07)	0.80 (0.72–0.90)	1.25 (1.14–1.37)
Generalised Circulatory Disturbances	743 (80.5%)	730 (81.2%)	428 (63.2%)	< 0.001	1.01 (0.92–1.10)	0.77 (0.66–0.91)	1.30 (1.16–1.46)
Tracheal Changes	663 (72.2%)	726 (81.4%)	401 (62.6%)	< 0.001	1.12 (1.04–1.20)	0.85 (0.75–0.97)	1.31 (1.16–1.47)
Subcutaneous Edema	776 (84.1%)	766 (85.2%)	459 (68%)	< 0.001	1.01 (0.95–1.06)	0.81 (0.72–0.91)	1.25 (1.13–1.37)
Muscular (skeletal) Discoloration ⁎⁎⁎	317 (34.2%)	360 (40%)	238 (35%)	0.133	1.15 (1.00–1.33)	1.03 (0.85–1.25)	1.12 (0.93–1.35)
Ascites	31 (3.4%)	68 (7.6%)	86 (12.7%)	< 0.001	2.22 (1.04–4.72)	3.81 (1.91–7.58)	0.58 (0.37–0.92)
Pericardial Serous Effusion	21 (2.3%)	40 (4.5%)	23 (3.4%)	0.067	1.75 (0.74–4.12)	1.39 (0.41–4.71)	1.26 (0.76–2.07)
Liver Cirrhosis	5 (0.5%)	17 (1.9%)	31 (4.6%)	0.001	3.25 (1.36–7.77)	8.31 (2.83–24.35)	0.39 (0.18–0.84)
**Leg Problems (overall)**	**83 (9%)**	**62 (6.9%)**	**112 (16.5%)**	**< 0.001**	**0.76 (0.42–1.39)**	**1.85 (1.05–3.27)**	**0.41 (0.26–0.64)**
Epiphysiolysis	51 (5.5%)	23 (2.6%)	45 (6.6%)	0.026	0.46 (0.22–0.95)	1.18 (0.56–2.49)	0.39 (0.20–0.78)
Femoral Head Necrosis	50 (5.4%)	22 (2.4%)	45 (6.6%)	0.041	0.45 (0.20–1.00)	1.21 (0.61–2.42)	0.37 (0.18–0.78)
Osteomyelitis	42 (4.5%)	17 (1.9%)	40 (5.9%)	0.029	0.41 (0.18–0.94)	1.28 (0.59–2.75)	0.32 (0.14–0.73)
Arthritis	22 (2.4%)	37 (4.1%)	77 (11.3%)	< 0.001	1.72 (0.57–5.22)	4.91 (1.78–13.60)	0.35 (0.18–0.67)
Tibial Dyschondroplasia	10 (1.1%)	49 (5.5%)	49 (7.2%)	< 0.001	4.79 (2.54–9.00)	6.40 (3.48–11.79)	0.75 (0.53–1.05)
**Renal Disorders (overall)**	**29 (3.1%)**	**36 (4%)**	**75 (11%)**	**< 0.001**	**1.28 (0.72–2.25)**	**3.57 (2.10–6.08)**	**0.36 (0.23–0.56)**
Nephropathy	35 (3.8%)	40 (4.4%)	86 (12.7%)	< 0.001	1.17 (0.63–2.19)	3.33 (2.01–5.51)	0.35 (0.21–0.58)
Uremic Muscle	13 (1.4%)	12 (1.3%)	43 (6.3%)	< 0.001	0.98 (0.35–2.72)	4.70 (2.21–9.99)	0.21 (0.09–0.48)
Renomegaly	11 (1.2%)	21 (2.3%)	27 (4%)	0.029	1.80 (0.69–4.72)	3.16 (1.15–8.68)	0.57 (0.36–0.91)
Gout	2 (0.2%)	2 (0.2%)	5 (0.7%)	0.247	1.09 (0.14–8.22)	3.82 (0.67–21.88)	0.29 (0.05–1.71)
Dehydration	26 (2.8%)	33 (3.7%)	74 (10.9%)	< 0.001	1.32 (0.68–2.58)	3.92 (2.20–6.97)	0.34 (0.20–0.56)
**Septicemia (overall)**	**52 (5.6%)**	**22 (2.4%)**	**38 (5.6%)**	**0.006**	**0.44 (0.26–0.76)**	**1.02 (0.64–1.61)**	**0.44 (0.26–0.73)**
Hepatomegaly	29 (3.1%)	49 (5.5%)	42 (6.2%)	0.082	1.65 (1.04–2.61)	1.91 (1.08–3.37)	0.86 (0.60–1.24)
Liver Necrosis	11 (1.2%)	8 (0.9%)	20 (3%)	0.009	0.77 (0.24–2.42)	2.55 (0.97–6.70)	0.30 (0.14–0.67)
Splenomegaly	3 (0.3%)	12 (1.3%)	22 (3.2%)	0.004	4.18 (0.46–37.95)	10.25 (1.45–72.40)	0.41 (0.19–0.88)
**Trauma**	**17 (1.8%)**	**39 (4.3%)**	**45 (6.6%)**	**0.008**	**2.33 (1.07–5.10)**	**3.57 (1.60–7.97)**	**0.65 (0.43–0.99)**
**Polyserositis (overall)**	**60 (6.5%)**	**16 (1.8%)**	**18 (2.7%)**	**< 0.001**	**0.28 (0.14–0.54)**	**0.40 (0.17–0.94)**	**0.70 (0.27–1.83)**
Pericarditis	63 (6.8%)	18 (2%)	21 (3.1%)	0.005	0.30 (0.14–0.63)	0.44 (0.20–0.98)	0.67 (0.25–1.79)
Perihepatitis	52 (5.6%)	15 (1.7%)	20 (2.9%)	0.002	0.30 (0.16–0.58)	0.51 (0.25–1.06)	0.59 (0.25–1.40)
Peritonitis	40 (4.3%)	17 (1.9%)	15 (2.2%)	0.029	0.45 (0.25–0.81)	0.49 (0.19–1.26)	0.91 (0.34–2.46)
Airsacculitis	46 (5%)	13 (1.4%)	9 (1.3%)	0.013	0.30 (0.12–0.77)	0.25 (0.09–0.73)	1.18 (0.37–3.78)
**Intestinal Volvulus**	**21 (2.3%)**	**15 (1.7%)**	**11 (1.6%)**	**0.875**	**0.71 (0.19–2.64)**	**0.70 (0.19–2.51)**	**1.02 (0.52–1.97)**
**Cannibalism and Pecking**	**9 (1%)**	**11 (1.2%)**	**19 (2.8%)**	**0.125**	**1.31 (0.37–4.60)**	**3.06 (0.53–17.71)**	**0.43 (0.19–0.96)**
**Non Specific Lesion**	**10 (1.1%)**	**9 (1%)**	**22 (3.2%)**	**0.007**	**-**	**-**	**-**
**Skin Lesions (overall)**	**43 (4.6%)**	**110 (12.2%)**	**152 (22.4%)**	**< 0.001**	**2.54 (1.58–4.10)**	**4.78 (3.10–7.37)**	**0.53 (0.34–0.83)**
Scratches and Wounds	25 (2.7%)	82 (9.1%)	72 (10.6%)	< 0.001	3.28 (1.70–6.30)	3.86 (1.86–8.00)	0.85 (0.45–1.61)
Cellulitis	7 (0.8%)	5 (0.6%)	16 (2.4%)	0.029	0.77 (0.18–3.27)	3.22 (1.05–9.92)	0.24 (0.07–0.80)
Bruises	9 (1%)	12 (1.3%)	8 (1.2%)	0.866	1.38 (0.50–3.76)	1.30 (0.32–5.28)	1.06 (0.31–3.69)
Hock Burn	2 (0.2%)	12 (1.3%)	61 (9%)	< 0.001	5.85 (1.10–31.17)	40.55 (8.90–184.85)	0.14 (0.06–0.37)
Footpad Dermatitis	2 (0.2%)	13 (1.4%)	19 (2.8%)	0.008	6.21 (2.06–18.73)	12.35 (2.11–72.29)	0.50 (0.14–1.76)

*p*-values were adjusted using the Benjamini–Hochberg procedure.

⁎ Indicates adjusted *p* < 0.001.

⁎⁎ Causes of mortality after the first week in bold.

⁎⁎⁎ Only discoloration associated with cardiovascular collapse was recorded, such as congestion, pallor with congestion, and generalized cyanosis.

Cardiovascular collapse was observed in all groups: 85.7% in the <18-day-old group, 86.3% in the 18-28-day-old group, and 68.3% in the ≥28-day-old group. Cardiovascular collapse and four related gross lesions were significantly more prevalent in birds younger than 18 days and in those aged 18-28 days than in birds aged 28 days or older. The likelihood of cardiovascular collapse, lung edema, general circulatory disturbances, tracheal changes, and subcutaneous edema was higher in birds aged under 18 days and 18-28 days than in those aged ≥ 28 days, with prevalence ratios and 95% CIs of 1.27 (1.12–1.43) and 1.27 (1.15–1.39), 1.25 (1.11–1.39) and 1.25 (1.14–1.37), 1.30 (1.10–1.52) and 1.30 (1.16–1.46), 1.18 (1.03–1.33), and 1.31 (1.16–1.47), and 1.23 (1.10–1.39) and 1.25 (1.13–1.37), respectively. In contrast, birds aged ≥ 28 days had a higher likelihood of cardiovascular collapse accompanied by cardiac dilatation than those under 18 days, with prevalence ratios and 95% CIs of 1.50 (1.08-2.08). In addition, birds aged ≥28 days were more likely to develop ascites and liver cirrhosis than younger groups, and the 18-28 days group had a higher likelihood than the <18 days group.

Impact of age groups on gross leg lesions: an overall leg disorder pattern was observed across all groups, with 9% in the <18 days category, 6.9% in the 18-28 days category, and 16.5% in the ≥28 days group. The most notable differences were overall leg disorders and their related gross lesions. The likelihood of overall leg disorders, epiphysiolysis, femoral head necrosis, osteomyelitis, and arthritis was higher in birds aged ≥28 days compared to those aged 18-28 days, with prevalence ratios and 95% CIs of 2.44 (1.56–3.85), 2.56 (1.28–5.00), 2.70 (1.28–5.56), 3.13 (1.37–7.14), and 2.86 (1.49–5.56), respectively. Additionally, the likelihood of overall leg disorders and arthritis was higher in birds aged ≥28 days than in those aged 18-28 days, with ratios of 1.85 (1.05–3.27) and 4.91 (1.78–13.60), respectively. Concerning tibial dyschondroplasia, both the 18-28 days and ≥28 days groups showed nearly a fivefold increase compared to the youngest group, with ratios of 4.79 (2.54–9.00) and 6.40 (3.48–11.79), respectively.

The impact of age groups on renal findings showed overall renal disorders across all groups, with prevalences of 3%, 5.5%, and 7.2%, respectively. The p-adjusted values indicated statistically significant differences in overall renal disorders, nephropathy, uremic muscle, renomegaly, and dehydration, with birds ≥28 days old more frequently exhibiting these findings than younger groups. The prevalence ratios were 3.57 and 2.78 for overall renal disorders, 3.33 and 2.86 for nephropathy, 4.70 and 4.76 for uremic muscle, 3.16 and 1.75 for renomegaly, and 3.92 and 2.94 for dehydration, respectively.

Regarding how age groups (<18 days, 18-28 days, and ≥28 days) were associated with septicemia, an overall septicemia pattern was observed across all groups, with proportions of 5.6% in the <18 days group, 2.4% in the 18-28 days group, and 5.6% in the ≥28 days group. Compared to the 18-28 days group, both the <18 days and ≥28 days groups showed higher overall septicemia, with prevalence ratios and 95% CIs of 2.27 (1.37–3.85) for each. Liver necrosis was more common in the ≥28 days group than in the 18-28 days group (PR=3.33, 95% CI: 1.49–7.14). The prevalence of splenomegaly was also higher in the ≥28 days group than in both the <18 days and 18-28 days groups, with prevalence ratios and 95% CIs of 10.25 (1.45–72.40) and 2.44 (1.14–5.26), respectively.

Among different age groups, trauma was observed in 1.8%, 4.3%, and 6.6% of each group, respectively. The prevalence was significantly higher in the 18-28 days and ≥28 days groups than in the <18 days group, with prevalence ratios of 2.33 (95% CI: 1.07–5.10) and 3.57 (95% CI: 1.60–7.97), respectively.

The influence of age groups on serosal membrane lesions indicates that polyserositis was present in 6.5%, 1.85%, and 2.7% of the groups, respectively. The occurrence of polyserositis and associated gross lesions was higher in birds <18 days than in those aged 18-28 days, with prevalence ratios and 95% CIs of 3.57 (1.85–7.14), 3.33 (1.59–7.14), 3.33 (1.72–6.25), 2.22 (1.23–4.00), and 3.57 (1.30–8.33). Additionally, the <18 days group had a higher prevalence of airsacculitis than the ≥28 days group (PR=4.00, 95% CI: 1.37–11.11).

### Skin lesions

A total of 306 dead birds had skin lesions that were not considered primary causes of death. Skin lesions accounted for 17.5% in Ross 308-HD, 6.6% in Ross 308-LD, and 12.3% in Rustic Gold birds (Table 3). Compared with Ross 308-LD, Ross 308-HD had a higher overall proportion of skin lesions (PR = 2.63, 95% CI: 1.47–4.55). The prevalence of scratches and wounds was significantly higher in the Rustic Gold birds than in the Ross 308-LD group (PR = 3.23; 95% CI: 1.75–6.25). Footpad dermatitis and hock burns were most prevalent in the Ross 308-HD group compared with the Rustic Gold and Ross 308-LD groups, with PR = 12.26 (1.40–106) and PR = 6.67 (95% CI: 1.39–33.33), respectively, for footpad dermatitis, and PR = 4.71 (1.35–16.42) and PR = 3.13 (95% CI: 1.32–7.69), respectively, for hock burns.

The impact of age group (birds <18 days, 18-28 days, and ≥28 days old) on skin lesions shows that the overall skin lesion pattern was present at 4.6%, 12.2%, and 22.4%, respectively (Table 4). Compared with the <18 days group, the ≥28 days group had a higher prevalence of overall skin lesions, scratches, cellulitis, hock burns, and footpad dermatitis, with prevalence ratios and CIs of 4.78 (3.10–7.37), 3.86 (1.86–8.00), 3.22 (1.05–9.92), 40.55 (8.90–184), and 12.35 (2.11–72), respectively. Compared with the <18 days group, the 18-28 days group had higher prevalence of overall skin lesions, scratches, hock burns, and footpad dermatitis, with prevalence ratios and CIs of 2.54 (1.58–4.10), 3.28 (1.70–6.30), 5.85 (1.10–31.17), and 6.21 (2.06–18.73), respectively. In addition, compared with those aged 18-28 days, the ≥28 days group had higher prevalence of overall skin lesions, cellulitis, and hock burns (PR = 1.89, 95% CI: 1.20–2.94), (PR = 4.17, 95% CI: 1.25–14.29), and (PR = 7.14, 95% CI: 2.70–16.67), respectively.

### Bacteriology

A total of 317 necropsied dead broilers were sampled for bacterial culture from at least one anatomical site, resulting in a total of 398 samples. Of these, 291 (73.12%) showed bacterial growth. Among the samples with bacterial growth, 241 were identified as species by MALDI-TOF. The six most common bacterial species identified across all organs of dead birds were *Escherichia coli* (47.7%), *Staphylococcus cohnii* (13.4%), *Enterococcus faecalis* (8.8%), *Staphylococcus aureus* (7.9%), *Staphylococcus lentus* (7.5%), and *Staphylococcus saprophyticus* (5.4%).

## Discussion

The main aim of this study was to determine whether Rustic Gold broilers exhibit lower mortality and a different pattern of causes of death and gross lesions in dead birds, with an emphasis on growth-related diseases under non-outbreak conditions, compared with Ross 308. The second aim was to investigate whether reducing the stocking density of Ross 308 influences the prevalence of the common causes of death.

The data showed no significant differences in mortality among birds older than 7 days across the three rearing systems. This finding is consistent with the findings of Guinebretière et al. (2024), who observed no significant difference in overall or late mortality (post-11 days of age) between Ross 308 and Rustic Gold. Furthermore, Guinebretière et al. (2024) reported that densities of 37 kg/m² (HD) and 29 kg/m² (LD) did not influence mortality in Ross 308. In contrast, Forseth et al. (2024) observed significant differences in mortality after the first week, with Ross 308 exhibiting higher average mortality proportions (3.05%) than Hubbard JA787 (1.49%). Similarly, van der Eijk et al. (2023) reported that fast-growing broilers (Ross 308) had a notably higher mortality proportion than slower-growing broilers (Ranger Classic), while stocking density did not significantly affect mortality. An explanation for these differences may be that the slower-growing strains investigated in these studies have considerably lower daily weight gain than Rustic Gold, underscoring the need to evaluate different broiler strains with varying growth rates (slow, slower, and fast).

In this study, a multivariable Poisson regression with a log link and cluster-robust standard errors was employed to produce unbiased risk ratios, accounting for flock dependence and potential confounders. This analytical approach provides more accurate and rigorous inferences than unadjusted models. The observed lack of significant differences in causes of death and gross lesions across strain and density in the results, compared with prior research, may partly be due to our analytical methods. Furthermore, variations in environment and management across flocks were considered. Together, these findings indicate that farm-level differences may more likely influence these effects than fundamental differences between systems.

From a commercial production standpoint, deviations from the highly efficient conventional broiler production system have significant economic consequences. Allowing 7 days for cleaning between rotations and rearing 41 kg/m^2^ in 34 days results in 8.9 rotations annually, while rearing for 41 days yields approximately 7.6 rotations per year. Rearing 36 kg/m^2^ of Rustic Gold birds over 41 days, or 33 kg/m^2^ of Ross 308 over 34 days, achieves about 75% and 81%, respectively, of the production level of 41 kg/m2 of Ross 308 over 34 days. Additionally, feed consumption is 12% higher in the Rustic Gold strain, and the price of day-old chickens is 15% higher than that of the Ross 308 strain. The economic impact of various welfare measures should be examined further to identify practical, cost-effective ways to improve animal welfare while maintaining food security.

### Cardiovascular collapse

This study found that over 80% of dead broilers on farms suffered from cardiovascular collapse, a condition linked to growth. This high proportion aligns with Schwean-Lardner et al. (2013), who noted that sudden death syndrome (SDS) and cardiac disease with ascites accounted for nearly fifty percent of the total mortality across different slaughter ages. Heart-related mortalities mainly occur in fast-growing broilers, with ascites and sudden death syndrome being the most common conditions (Olkowski, 2007). Data showed that most broilers with cardiovascular collapse also exhibited lung edema/lung congestion, subcutaneous edema, tracheal changes, and generalized circulatory disturbances. This does not align with Kittelsen et al. (2015), who noted that most broilers exhibited lung congestion; however, no other lesions were observed throughout the body.

Our data allow us to distinguish between cardiac failure caused by heart dilatation and the condition often referred to as SDS, which is considered a metabolic condition. Gross lesions such as heart dilatation, ascites, liver fibrosis, pericardial effusion, and subcutaneous edema indicate chronic stages of cardiovascular collapse (Olkowski and Classen, 1998; Olkowski, 2007). In contrast, congestion and lung edema, generalized circulatory disturbances, and muscle discoloration are usually associated with a more sudden cardiovascular collapse. Kittelsen et al. (2015) proposed that a possible cause of lung congestion (congested and edematous lungs, possibly accompanied by mottled red and white pectoral muscles, and possibly involving congestion of the liver and spleen) is SDS.

This study also found that the likelihood of cardiovascular collapse and associated gross lesions was higher in the Ross 308 groups than in Rustic Gold birds. Nonetheless, the higher significant differences were observed only in the cardiovascular collapse condition accompanied by cardiac dilatation (prevalence ratio >1.7) in both Ross 308 groups compared with Rustic Gold birds. This cardiovascular collapse condition was associated with gross lesions, including ascites, liver cirrhosis, pericardial serous effusion, and subcutaneous edema. The magnitude of these effect estimates, along with their reasonably precise confidence intervals, increases confidence that these associations are biologically meaningful rather than random variation. Because the cardiovascular collapse condition with cardiac dilatation is recognized as an indicator of impaired cardiovascular function and compromised welfare (Olkowski et al*.*, 2020; Forseth et al*.*, 2023), these findings suggest that Ross 308 broilers, despite having shorter lifespans than Rustic Gold broilers, are more prone to chronic cardiovascular disorders under commercial production conditions. High body weight and daily gains have previously been associated with high ascites-related mortality, making up nearly 50% of total mortality (Arce-Menocal et al*.*, 2009; Kalmar et al*.*, 2013). Ascites in broiler chickens results from an imbalance between oxygen supply and the oxygen required to support rapid growth and high feed efficiency (Decuypere et al*.*, 2000). This has been attributed to decreased cardiac output, either due to the heart's smaller muscle mass relative to body weight or to significantly increased metabolic oxygen demand during rapid growth, thereby increasing the risk of tissue hypoxia. Additionally, impaired pulmonary function leads to relative hypoxia within the body (Guo et al*.*, 2023). Hypoxia triggers compensatory mechanisms in the cardiopulmonary system, including remodeling of the pulmonary arteries and right ventricular hypertrophy (Chen et al*.*, 2025). In cardiopulmonary tissues, cells switch from oxygen-dependent metabolism (aerobic respiration) to anaerobic glycolysis to continue producing ATP (Stenmark et al., 2015) and to form oxygen-free radicals (Veit et al*.*, 2015). Pulmonary hypertension can lead to right heart failure, which in turn increases central venous pressure, causes fluid buildup in body cavities, and enlarges the right ventricle in weight and volume (McGovern et al*.*, 1999). All broilers with ascites experience severe hypoxia, but many fast-growing broilers have been shown to be hypoxic without developing ascites (Olkowski, 2007). The normalized heart weight decreased over time in both the slow-growing strain (Illinois) and the fast-growing Ross strain, but only the Ross strain showed a significant decrease (Zhang et al*.*, 2018).

Regarding age group analysis, the probability of chronic cardiovascular collapse—such as heart dilatation, ascites, and liver cirrhosis—showed an increase with older age. Conversely, cardiovascular collapse without cardiac dilatation became less common with increasing age. This aligns with Grafl et al. (2020), who reported positive correlations between ascites and cardiac changes, such as dilatation and hypertrophy, and that the incidence of ascites was significantly higher at the fifth week of age than at the second week in Ross 308 broilers. This is reasonable because, as birds age, there is more time for the development of chronic cardiovascular collapse, one of the most common gross lesions, which includes cardiac dilatation. Even arrhythmia incidence has been shown to increase with age, reaching 17% in the broiler population at 42 days, and cardiac arrhythmias not only play a role in the pathogenesis of sudden death syndrome but may also be part of the development of ascites in some cases (Olkowski and Classen, 1998).

Approximately 75% of mortality in Rustic Gold birds was due to cardiovascular disease. Although this is lower than in Ross 308-HD (86%), it is still a very high proportion and may be explained by the relatively high daily weight gain of this strain (49-53 grams), which is not very different from the weight gain of Ross 308 (61-63 grams). It would be relevant to determine how low the daily weight gain should be in a particular strain before, for example, a 50% reduction in this condition can be achieved. What is the level of cardiovascular disease that is acceptable in broiler production?

### Leg disorders

The current findings show that more than 10% of broilers that died or were euthanized on farms had leg disorders, which aligns with Grafl et al. (2020), who reported that lesions in the femoral head were associated with a higher risk of death during the 7–28 day period. It has been reported that mortality caused by lameness can range from 5% to 10% (Dinev et al*.*, 2012). Leg disorders as a cause of mortality are mainly due to the weakness of birds suffering from such conditions, resulting in, e.g., kidney failure with subsequent uremia and lack of feed. Poor locomotion was previously shown to be present in more than 27.6% of birds having gait scores of 3 or higher (24.3%, 3%, and 0.2%, respectively), of which 3.3% of the birds were nearly unable to walk, falling into gait scores 4 and 5 (3.1% and 0.2%, respectively) (Knowles et al*.*, 2008).

Bacterial chondronecrosis with osteomyelitis presents a significant challenge to broiler health and welfare, with potential severe impacts on meat quality, production, food safety, and economic losses (Anthney et al*.*, 2024). The data showed no significant differences in either overall leg disorder occurrence across production types or single-gross lesion observations among them. These findings do not align with previous observations by Riber and Wurtz (2024), Damaziak et al. (2019), and Guinebretière et al. (2024), who reported that strains with rapid growth tend to exhibit poor leg health and impaired locomotion, which may affect their ability to perform behaviors and access resources. Baxter et al. (2021) reported that gait scores were significantly lower in slow-growing broilers (Hubbard Redbro) than in Ross 308. However, we did not observe gait in the current study. Fast-growing broilers at weeks 3 and 5 increase body weight by 16- and 36-fold, compared to 1-day-old chicks, whereas Slow-growing broilers increase by 13- and 26-fold. This does not sufficiently enlarge the femora and tibiae, thereby increasing leg strain, especially in fast growers (Damaziak et al*.*, 2019). However, we cannot explain the discrepancies between earlier studies and our findings, except to suggest that variations in broiler strains or differences in how leg health was assessed might account for them.

Significant differences were observed across all leg disorders by age group, with the highest likelihood in the ≥ 28-day-old group. These results align with gait scores, which generally worsened from the fourth to sixth weeks (Baxter et al*.*, 2021). It could be speculated that birds with leg disorders have a higher risk of dying at older ages because lameness severity increases with age, limiting access to food and water and leading to culling or death. These findings partially align with existing research on arthritis (Grafl et al*.*, 2020), which reported that the detection of joint lesions was positively associated with broiler weight. Regarding TD, the significantly higher prevalence among older broilers is inconsistent with Amini et al. (2026), who reported that TD incidence decreased with age (*p* < 0.0001) in broilers.

### Skin lesions

Although skin lesions are not considered a cause of mortality in themselves, they may be considered a welfare problem and a major economic problem due to condemnation. The overall occurrence of skin lesions was 305 (12.2%), and scratches and wounds accounted for more than 60% of cases. The overall skin lesions, 'scratches and wounds', hock burns, and footpad dermatitis varied significantly between bird groups. Ross 308-HD exhibited a 3- to 6-fold higher prevalence than Ross 308-LD. In addition, the prevalence of scratches and wounds was three times higher in Rustic Gold than in Ross 308-LD. These differences are biologically important because skin lesions negatively affect broiler welfare by causing pain, compromising skin integrity, and increasing the risk of secondary bacterial infections (Schulze Bernd et al., 2020; Nielsen et al., 2023). The higher prevalence observed in Ross 308-HD suggests a less favorable skin-welfare profile under commercial production conditions. Because the absolute prevalence of hock burns and footpad dermatitis remained low, particularly in Rustic Gold and Ross 308-LD, the practical importance of these differences should be interpreted with caution. Similar results were reported by van der Eijk et al. (2023), who found that a stocking density of 42 kg/m² differed significantly from lower densities (24, 30, 36 kg/m²) in the number of skin lesions, indicating a decline in skin health with increasing stocking density. However, Sztandarski et al. (2025) reported that reducing stocking density from 41 kg/m² to 35 kg/m² in Ross 308 chickens did not affect the prevalence of skin injuries. Our findings are not in accordance with those of van der Eijk et al., 2023), who showed that slow-growing broilers (Ranger Classic) had fewer skin lesions than fast-growing broilers (Ross 308). In our results, we did not observe significant differences in scratches and wounds in Ross 308 at a high stocking density and Rustic Gold. This does not align with de Jong et al. (2022), who reported scratches and wounds were significantly more common in slower-growing broilers (Dutch Retail Broiler, with a stocking density of 38 kg/m^2^ and daily weight gain of 50 g) compared to fast-growing broilers (which grow over 60 g/day and are housed at a maximum density of 42 kg/m^2^).

Regarding hock burns and footpad results, it is comparable with the results of van der Eijk et al. (2023), who reported that slow-growing broilers had lower prevalences of hock burns and footpad dermatitis than fast-growing broilers, and that low densities resulted in lower footpad dermatitis scores than high densities. A study by de Jong et al. (2022) compared fast-growing with high-density versus slower-growing broilers and reported that both scores of hock burns and footpad dermatitis were significantly higher in conventional broilers. This seems reasonable, as Ross 308 has a higher body weight and engages in fewer prolonged activity bouts than the slower-growing breed (Hubbard Redbro) (Baxter et al., 2021). Guinebretière et al. (2024) reported that Rustic Gold birds were more active, more likely to stand, and more likely to interact with the enrichments than Ross 308 birds. Additionally, high density and high litter moisture levels increase microbial activity, leading to higher temperatures and ammonia levels in broiler houses, which can consequently result in higher rates of contact dermatitis (Bessei, 2006). Despite the addition of extra litter material during rearing and a shorter rearing period, the litter quality of a Ross 308 group was poorer than that of a Rustic Gold group, and the gait score was higher in the Ross 308 (Guinebretière et al*.*, 2024). Shynkaruk et al. (2023) reported that average footpad scores and litter moisture consistently decreased as stocking densities decreased (from 41.5 to 31 kg/m²). Furthermore, the increased stocking density (41 kg/m²) compared to a lower density (35 kg/m²) in Ross 308 chickens was reported to be associated with higher FPD scores, while no significant difference was observed concerning hock burns (Sztandarski et al*.*, 2025). However, we did not assess the litter quality in this study.

The findings showed that overall skin lesions, including scratches, cellulitis, hock burn, and footpad dermatitis, increased in broiler farms as the birds aged. This is in accordance with Škrbić et al. (2015), who reported that Footpad dermatitis and hock burns were significantly more prevalent at 42 days than at 21 days, and Opengart et al. (2018), who reported that lighter broilers exhibited significantly lower levels of footpad dermatitis and gait scores than heavier broilers. Meaning that as birds get older, size increases and mobility declines, leading to longer sitting times, pressure on hocks and breast, and more lesions (Riber and Wurtz, 2024). Our results showed that both leg disorders and skin lesions increased with age in the birds. Leg disorders impair mobility and lead to a staggering gait, increasing the risk of hitting farm objects. This can cause scratches that might develop into cellulitis (Norton et al*.*, 1999). Sitting for long periods on litter can cause hock burns and footpad dermatitis (Riber and Wurtz, 2024).

### Renal disorders

Rustic Gold broilers had a higher prevalence of nephropathy, dehydration, and uremic muscle lesions than fast-growing broilers. These conditions likely represent different manifestations of the same underlying pathological process rather than independent conditions. In birds, dehydration reduces glomerular filtration, leading to urate buildup in the renal tubules. Prolonged or severe dehydration can cause tubular degeneration and renal failure (Phalen, 2020). Because this study was observational, the temporal relationship among dehydration, nephropathy, and uremic muscle lesions could not be determined.

The reason for the higher prevalence of renal lesions in Rustic Gold broilers remains unclear. One possible explanation is that the longer production cycle of this slower-growing strain results in greater cumulative exposure to environmental, nutritional, or metabolic factors that could harm kidney health. Behavioral differences may also play a role, as slower-growing broilers tend to be more active, engaging in more locomotion and foraging while spending less time feeding and drinking than faster-growing strains (Dixon, 2020). In more active flocks, smaller or sick birds may be at a disadvantage when competing for feeders and drinkers, possibly leading to reduced water intake and dehydration, which could contribute to increased renal lesions. To our knowledge, the pathophysiological mechanisms underlying strain-related differences in renal lesions between slow- and fast-growing commercial broilers have received little attention in the published literature. Further studies integrating gross pathology, histopathology, and serum biochemical analyses are necessary to better understand why slow-growing broilers have a higher prevalence of renal lesions.

### Trauma

The results indicated no significant differences in trauma between the Rustic Gold and Ross 308 groups. However, the prevalence of trauma increased significantly with age, possibly due to crowding on the farm and declining walking ability as birds grow older (*p* < 0.001) (Rasmussen et al., 2022). As broilers age, body weight increases more rapidly than skeletal strength (Damaziak et al., 2019). Additionally, heavier birds tend to produce higher impact forces during slips, collisions, or piling. Our findings showed trauma occurred in 4% of cases, which aligns with Kittelsen et al. (2015), who reported liver rupture, leading to intra-abdominal hemorrhage.

### Polyserositis

Our data showed a higher prevalence of polyserositis and associated lesions in Rustic Gold. However, only peritonitis showed a significant difference. Additionally, younger birds exhibited more polyserositis and related lesions compared to older birds. It could be speculated that Rustic Gold birds, or the youngest birds regardless of strain, tend to be more active than Ross 308 or older birds, and the litter tends to be drier. As a result, the house's air becomes dustier, which is known to carry bacterial contaminants such as *E. coli* (Nguyen et al*.*, 2022). Air sacs and lungs may serve as entry points for *E. coli,* allowing bacterial entry via damaged epithelium, and bacteria can be isolated from the liver in commercial broilers with moderate to severe lesions of airsacculitis, pericarditis, and perihepatitis (Pourbakhsh et al*.*, 1997). In a recent study of Ross 308 breeders vaccinated against E. coli, polyserositis was found in more than 7% of their progeny from 4 to 11 days of age (Kromann et al., 2022). To our knowledge, no previous studies have compared polyserositis among fast- and slow-growing broilers, across densities, or within age groups.

### Bacterial pathogens

Bacteriological examination was performed only on dead birds with gross lesions suggestive of bacterial infection, representing approximately 12% of the nearly 2508 necropsied broilers. The remaining birds were diagnosed at necropsy with predominantly non-infectious causes of death, including cardiovascular collapse, renal disorders, trauma, and other conditions, and therefore were not considered to require bacteriological examination. Among the bacteriologically examined birds, *Escherichia coli* was the most common bacterium (47.7%), followed by *Staphylococcus cohnii* (13.4%), *Enterococcus faecalis* (8.8%), *Staphylococcus aureus* (7.9%), *Staphylococcus lentus* (7.5%), and *Staphylococcus saprophyticus* (5.4%). The dominance of *E. coli* aligns with its established role as the primary bacterial pathogen in avian colibacillosis, including polyserositis, septicemia, osteomyelitis, omphalitis, and yolk sac infection (Nolan et al., 2017). In Danish broilers aged ≥ 21 days, *E. coli* has similarly been identified as the predominant pathogen associated with outbreaks of *E. coli*, with significantly higher frequencies of pericarditis, perihepatitis, airsacculitis, peritonitis, arthritis, and femoral head necrosis than in non-outbreak flocks (Kromann et al., 2023). *E. coli* is genetically diverse, and numerous sequence types have been associated with sporadic cases of avian colibacillosis (Kromann et al., 2023). Our results regarding *Staphylococcus cohnii* align with those of Szafraniec et al*.* (2025), in which, among all staphylococcal species identified, Staphylococcus cohnii was the most prevalent. Our results showed that *E. coli* isolates were most frequently found in the liver (53%) and in bone marrow and joints (36%). This is consistent with Grafl et al. (2020), who found that *E. coli* was detected more often in the liver than in the bone marrow at both the second and fifth weeks of age. In contrast, most isolates of *Staphylococcus spp*. (90%) were present in nearly equal proportions in the bone marrow and joints, according to our findings. This contradicts previous findings that staphylococcal leg infections in broilers predominantly affected the femur and femoral head (necrosis), with knee joint involvement being uncommon (Szafraniec et al., 2025). These findings show that *E. coli* remains the primary bacterial pathogen in birds with suspected infections, confirming avian colibacillosis as the principal bacterial disease, even though it accounts for only a small part of overall flock mortality from 7 days to slaughter age.

## Conclusion

To the authors' knowledge, this is the first major investigation into the specific causes of death and associated gross lesions in a slow-growing broiler strain, and its subsequent comparison with Ross 308 at different stocking densities. There were no significant differences in total mortality from 7 days post-hatch to slaughter, nor in the prevalence of the leading causes of death, including cardiovascular collapse, leg disorders, renal disorders, septicemia, trauma, polyserositis, cannibalism, and intestinal volvulus, across production types. However, several important gross lesions differed significantly. Chronic cardiovascular lesions, including ventricular dilatation, ascites, and liver cirrhosis, were less prevalent in Rustic Gold broilers and in birds younger than 18 days. By contrast, Ross 308 reared at the higher stocking density and birds ≥28 days of age had higher prevalences of footpad dermatitis, hock burns, and scratches.

Regarding strains and stocking densities, the results for mortality, leg disorders, and, to some extent, cardiovascular collapse differ from those of most previous research. This suggests a possible interaction between environmental and/or management factors and the statistical model used in this study, which separates the effects of the production type from those of farm management. Additionally, previous studies involve different strains of slower-growing birds that differ from the one examined here. These studies have not primarily focused on gross lesions, emphasizing the need for additional research.

Alternative broiler production types should be evaluated in the context of both animal welfare and production efficiency. Although slower-growing broilers may reduce the prevalence of certain gross lesions associated with impaired welfare, they also result in longer production cycles and higher production costs. A comprehensive evaluation that integrates pathological, production, welfare, and economic outcomes is therefore required to determine the overall sustainability of different broiler production types.

### Limitations

Because the study population comprised only dead birds, the results estimated prevalence ratios for causes of death among them rather than the risk of death across the entire farm population. Additionally, some dead birds had multiple causes of death; therefore, the total proportion across all causes or production types exceeded 100%. Although the models were adjusted for sex and season and accounted for flock-level clustering, the observational design does not permit causal inference regarding the effects of production type, and residual confounding from unmeasured factors may remain. Comparing Rustic Gold and Ross 308 requires caution because their production types differ in genetics, slaughter age, growth rate, and other commercial characteristics. Thus, the observed pathological differences cannot be attributed solely to genetics.

Additionally, since the study relied solely on postmortem examinations of broilers, welfare indicators for live birds—such as gait, mobility, and behavioral assessments—were not assessed. Furthermore, flock-level production and management factors, including feed conversion and litter quality, were outside the scope of this commercial field study and therefore not included in the current analysis.

## Declaration of AI and AI-assisted technologies in the writing process

During the preparation of this work, the authors used ChatGPT to help modify computational statistical code during analysis and Grammarly to occasionally improve the readability of the writing.

## Author contributions

**Jens P. Christensen** initially developed the overall study design with **Ida C.N. Thøfner, Susanne Kabell**, and **Tahseen A. AL- Saeedi**. All authors contributed intellectually to developing the specific research protocol, including defining the study population and farm eligibility criteria, developing the sampling strategy, and establishing the analytical framework. **Jens P. Christensen, Ida C.N. Thøfner**, and **Tahseen A. AL- Saeedi** conducted the postmortem examinations and contributed to the diagnostic assessment of the pathological findings. **Tahseen A. AL- Saeedi** and **Søren S.Nielsen** conducted the statistical analyses. **Tahseen A. AL- Saeedi** prepared the initial draft of the manuscript. **Jens P. Christensen, Ida C.N. Thøfner, Tahseen A. AL- Saeedi, Søren S.Nielsen**, and **Susanne Kabell** contributed to the scientific evaluation and interpretation of the results.

## Disclosures

The authors reported no potential conflict of interest.
